# Supplementary material for: Structural characterization of a novel human adeno-associated virus capsid with neurotropic properties
Source: Nat Commun. 2020 Jun 30;11:3279. doi: 10.1038/s41467-020-17047-1 (PMC7327033; doi:10.1038/s41467-020-17047-1)
Supplement: Supplementary file 1 — Supplementary Information [file 41467_2020_17047_MOESM1_ESM.pdf]

Supplementary Table 1.

Virus titer after large-scale production and purification

Vector name	Titer GC/mL
ssAAV2-*CB6-FLuc*	1.60E+12
ssAAVv66-*CB6-FLuc*	6.00E+12
ssAAVv66-Q39K-*CB6-FLuc*	7.70E+12
ssAAVv66-A151V-*CB6-FLuc*	9.00E+12
ssAAVv66-K447R-*CB6-FLuc*	6.60E+12
ssAAVv66-A450T-*CB6-FLuc*	8.80E+12
ssAAVv66-M457Q-*CB6-FLuc*	1.00E+13
ssAAVv66-A492S-*CB6-FLuc*	1.40E+13
ssAAVv66-D499E-*CB6-FLuc*	9.00E+12
ssAAVv66-Y533F-*CB6-FLuc*	7.10E+12
ssAAVv66-D546F-*CB6-FLuc*	1.20E+13
ssAAVv66-G548E-*CB6-FLuc*	7.80E+12
ssAAVv66-S585R-*CB6-FLuc*	7.00E+12
ssAAVv66-T588R-*CB6-FLuc*	1.30E+13
ssAAVv66-T593A-*CB6-FLuc*	8.10E+12
ssAAVv66-S585R/T588R-*CB6-FLuc*	1.85E+12

Supplementary Table 2. Cryo-EM data collection refinement and validation statistics

	#1 name (EMDB-20630) (PDB 6U3Q)
• Data collection and processing	
Magnification	47,214
Voltage (kV)	300
Electron exposure (e–/Å^2)	48.62
Defocus range (μm)	0.4-5.0
Pixel size (Å)	1.059
Symmetry imposed	I
Initial particle images (no.)	52,874
Final particle images (no.)	52,874
Map resolution (Å)	2.46
FSC threshold	0.143
• Refinement	
Initial model used (PDB code)	1LP3
Model resolution (Å)	2.6
FSC threshold	0.5
Map sharpening *B* factor (Å^2)	32.92
Model composition	
Non-hydrogen atoms	248,280
Protein residues	31,140
Ligands	0
B factors (Å^2)	
Protein	86.48
Ligand	0
r.m.s. deviations	
Bond lengths (Å)	0.009
Bond angles (°)	0.603
Validation	
MolProbity score	1.66
Clashscore	2.25
Poor rotamers (%)	4.15
Ramachandran plot	
Favored (%)	96.91
Allowed (%)	4.15
Disallowed (%)	0.00

Supplementary Table 3. Distances Measurements between atoms of interest from **Figure 6c and f**

Structure chain	A.A.#	Atom ID	Distance (Å)	Atom ID	A.A.#	Structure chain
v66 A	D499	OD1	5.2	NZ	K447	v66 3
v66 A	D499	OD1	3.8	OG1	T448	v66 3
1lp3 A	E499	OE1	2.2	OG1	T448	v66 3
1lp3 A	E499	OE2	3.5	NZ	K447	v66 3
1lp3 A	E499	OE2	2.8	N	T448	v66 3
1lp3 A	R585	N	3.1	O	R588	1lp3 A
1lp3 A	R585	O	2.7	N	R588	1lp3 A
1lp3 A	R585	O	3	N	N587	1lp3 A
1lp3 A	R585	NE	2.6	OD1	N587	1lp3 A
1lp3 A	S498	O	2.3	OG1	T448	v66 3
v66 A	S498	O	1.9	OG1	T448	v66 3
v66 A	S585	N	2.9	O	T588	v66 A
v66 A	S585	O	3.2	N	T588	v66 A
v66 A	S585	O	3.1	N	N587	v66 A

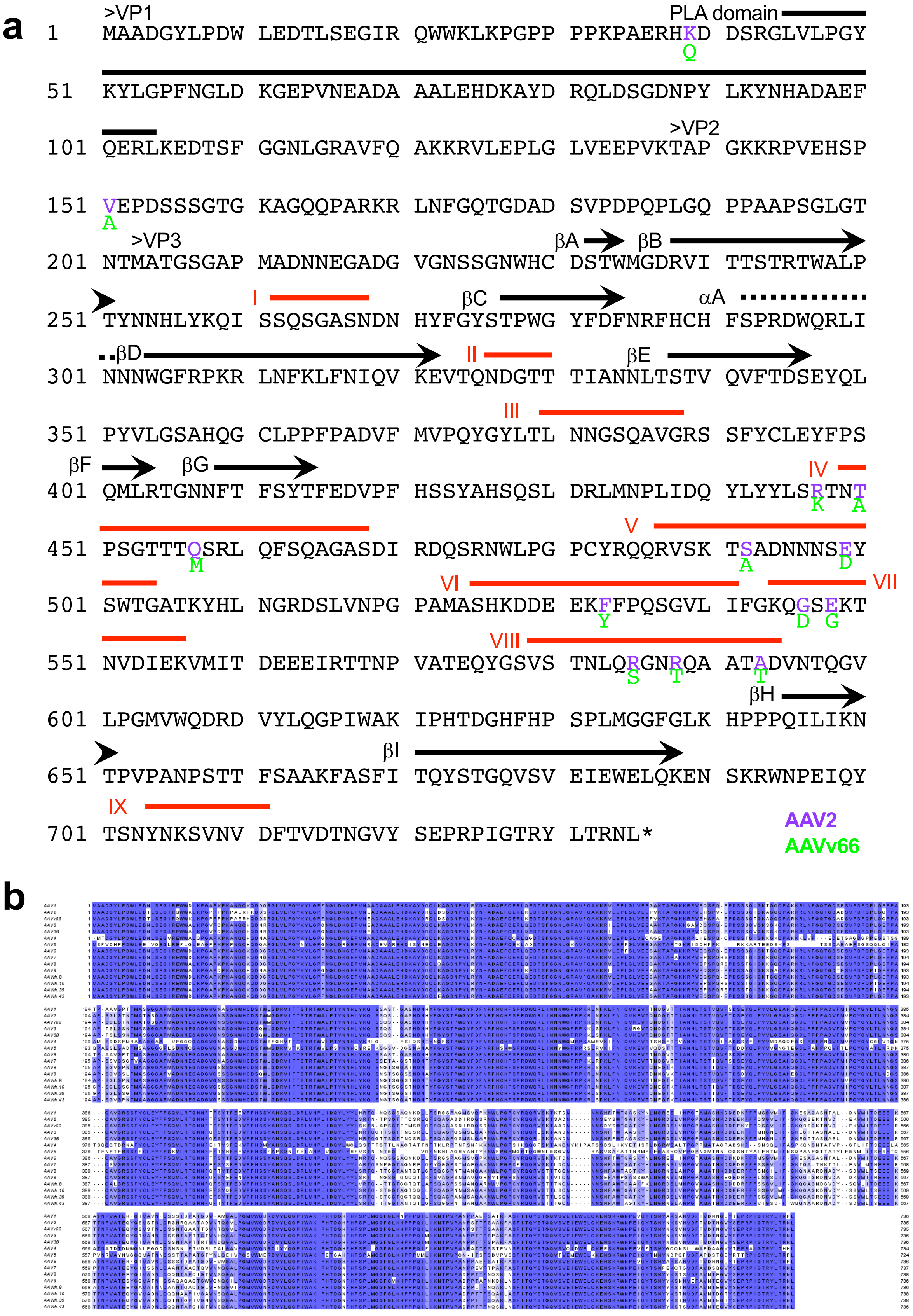

Supplementary Figure 1. Amino acid sequence of the AAVs/AAVv66 capsid. (a) Amino acid differences between AAV2 (purple) and AAVv66 (green) are highlighted. Variable region (VR) residues are denoted by red bars. The αA domain is demarcated by the dotted bar, and residues forming the β-sheets are marked with black arrows. Start positions for VP1, VP2, and VP3 are marked by greater-than symbols (>). The PLA domain within VP1 is denoted by a black bar. **(b)** Amino acid difference between AAVv66 and AAV1-9. Blue shaded amino acids, >80% similarity; medium-blue, >60% similarity; light-blue, >40% similarity, dashes; gaps.

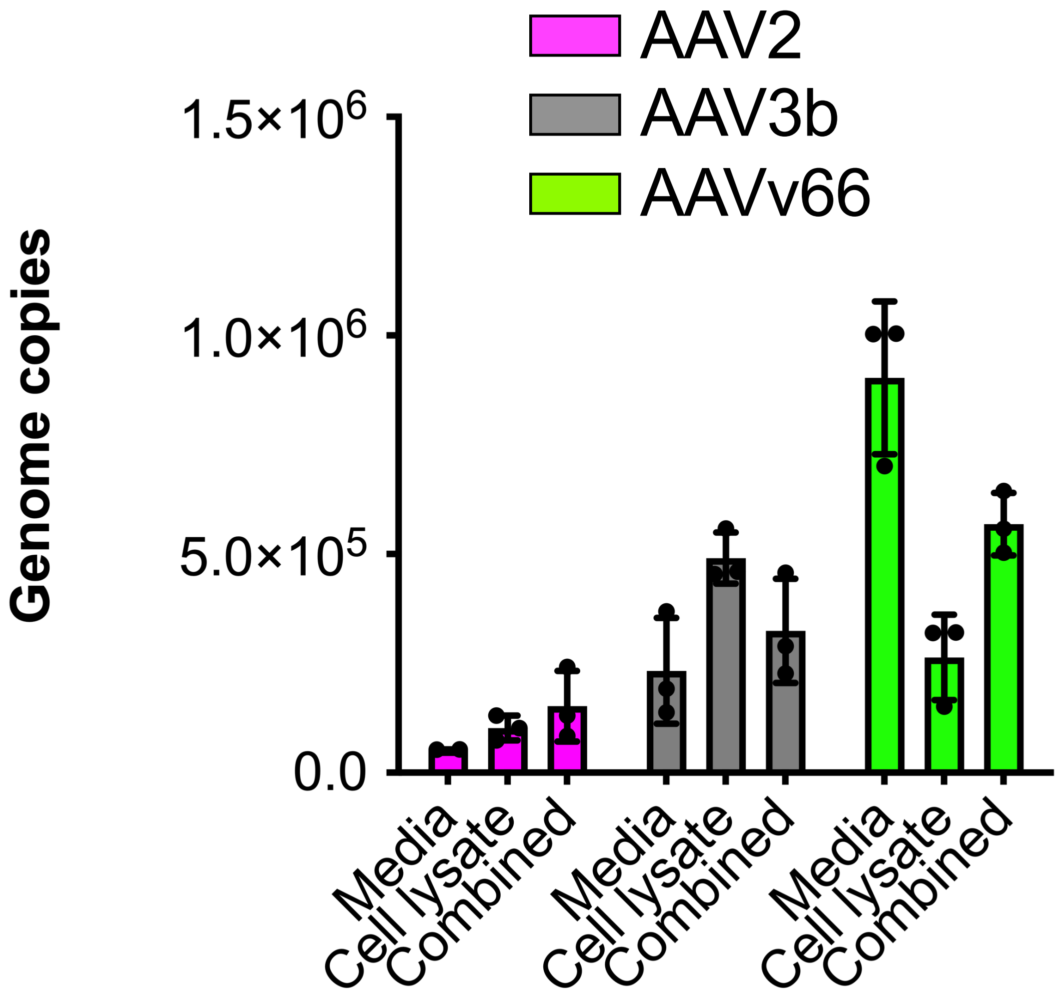

Supplementary Figure 2. AAVv66 produces higher vector yields than AAV2. Crude lysate PCR assays were performed on media and cellular lysates of HEK239 cells subjected to triple-transfection of pAAV and packaging plasmids for AAV2 (magenta bars) or AAVv66 (green bars). Combined values represent yields obtained from both media and cell lysate material pooled together. Values represent mean genome copies ±SD, *n* = 3/group.

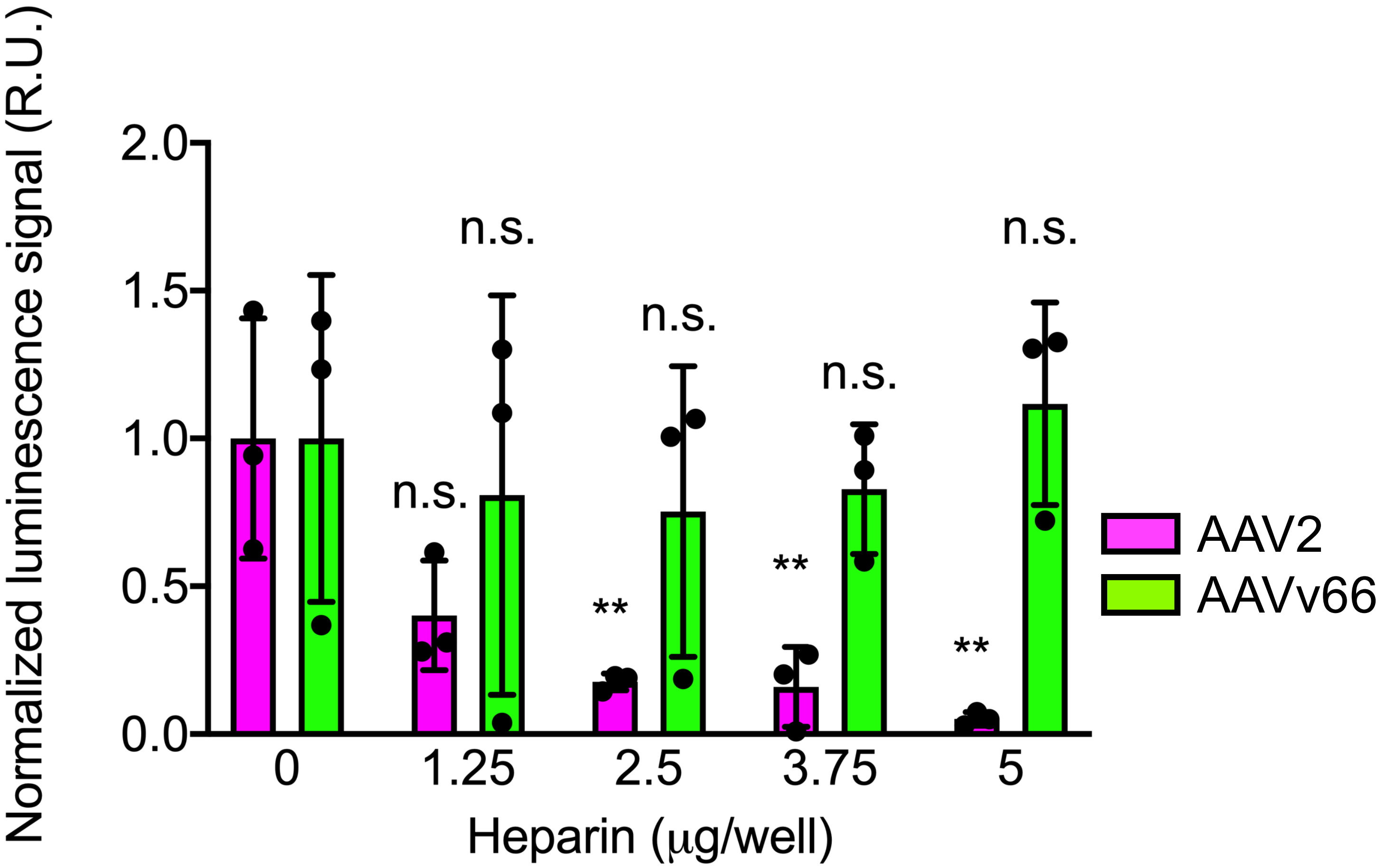

Supplementary Figure 3. **AAVv66 lacks strong heparin binding.** Heparin competition assay showing transduction efficiency of AAV2-*CB6-Fluc* (magenta bars) and AAVv66-*CB6-Fluc* (green bars) in HEK293 cells in the presence of increasing amounts of heparin (x-axis). Luminescence values were scaled to values obtained for wells lacking heparin and set to 1 (y-axis). Values represent mean ±SD, n = 3/group, **p<0.01 by two-way ANOVA.

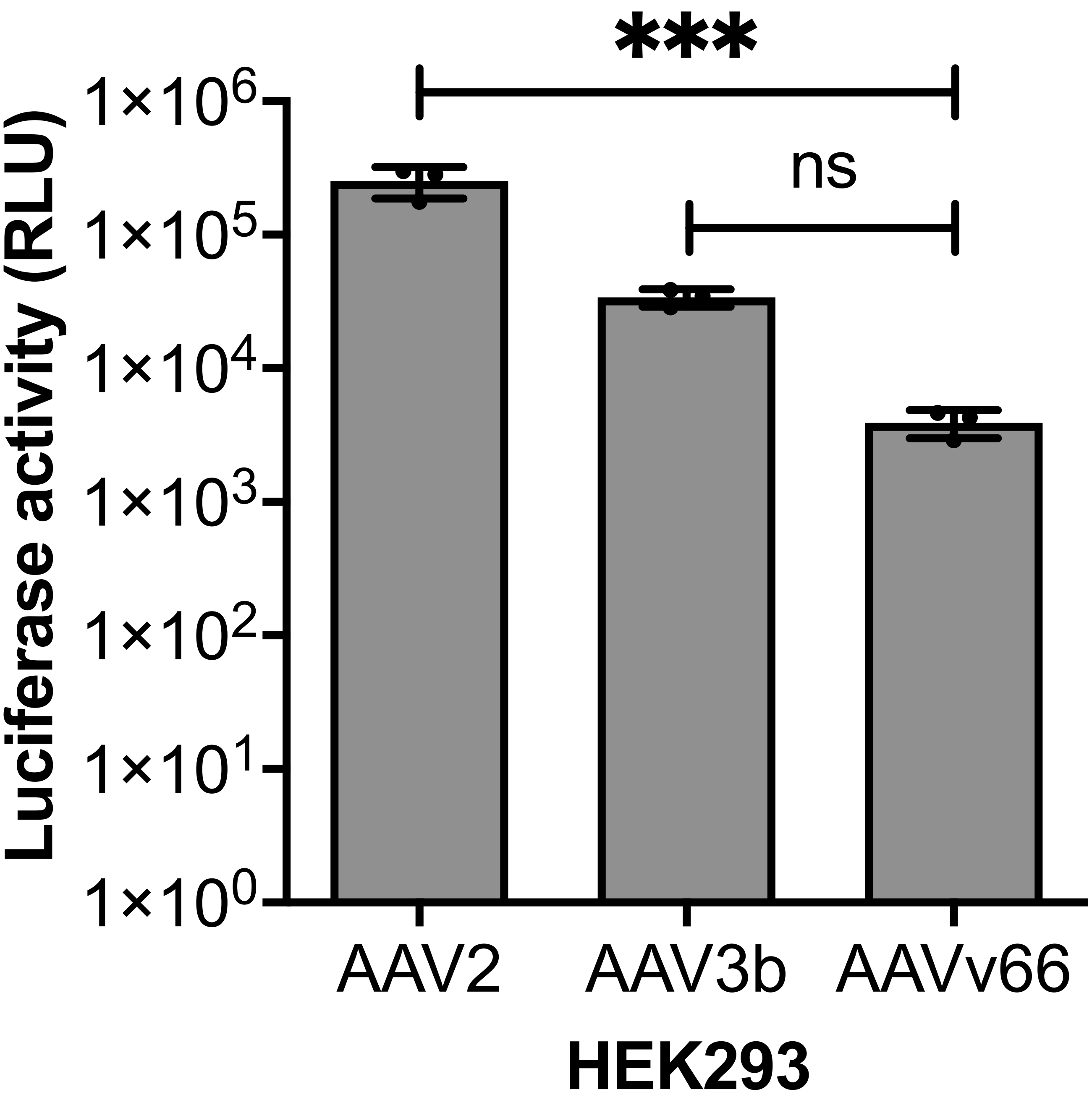

Supplementary Figure 4. ***In vitro* infection efficiencies of AAV2, AAV3b, and AAVv66 in HEK293 cells.** Vectors were packaged with *CB6-Fluc*. Cells were lysed 48-hr post-infection to assess the infectivity of vectors via detection of luciferase activity (RLU, relative light units). Data is displayed in log-scale. Values represent mean ±SD, ****p*<0.0001 by one-way ANOVA, *n* = 3/group.

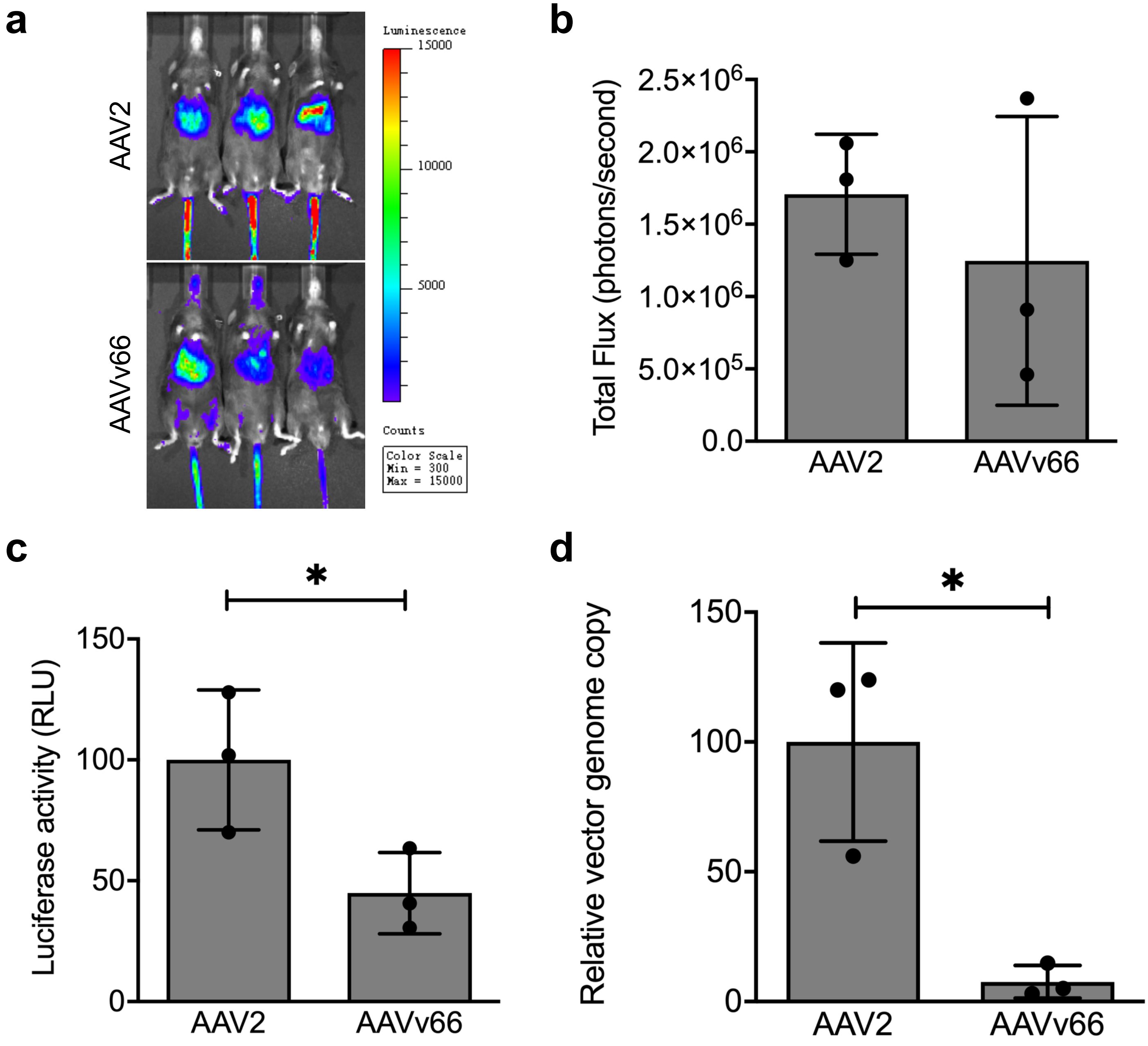

Supplementary Figure 5. Intravenous administration of AAVv66 vector shows transduction of the liver. Systemic injection of AAVv66-*CB6-PI-Fluc* resulted in the predominant transduction of the liver. rAAV2-*CB6-PI-Fluc* or AAVv66-*CB6-PI-Fluc* (1.0E11 GC per mouse) was injected into mice by tail vein administration. **(a)** After 14 days, mice were injected with luciferin substrate intraperitoneally and imaged on the ventral side. Although quantification of whole-body live bioluminescence of luciferase activity did not reveal significant differences in transduction of the liver between AAVv66-*CB6-PI-Fluc* and AAV2-*CB6-PI-Fluc*, isolation of liver tissues and quantification of luciferase activity and detection of vector genome copy by qPCR showed that AAVv66 is a significantly weaker transducer of liver than AAV2. In addition, AAVv66 shows some luminescence from the jaw/snout area. Scale bar represents luminescence (flux, photons/sec) **(b)** Total flux of the abdomen in acquired images (**panel a**) was recorded. Tissues were harvested and assayed for luciferase activity **(c)** and vector genome abundance by qPCR **(d)**. Values represent mean ±SD, n = 3 mice/group, *p <0.05 by two-tailed Student's t test.

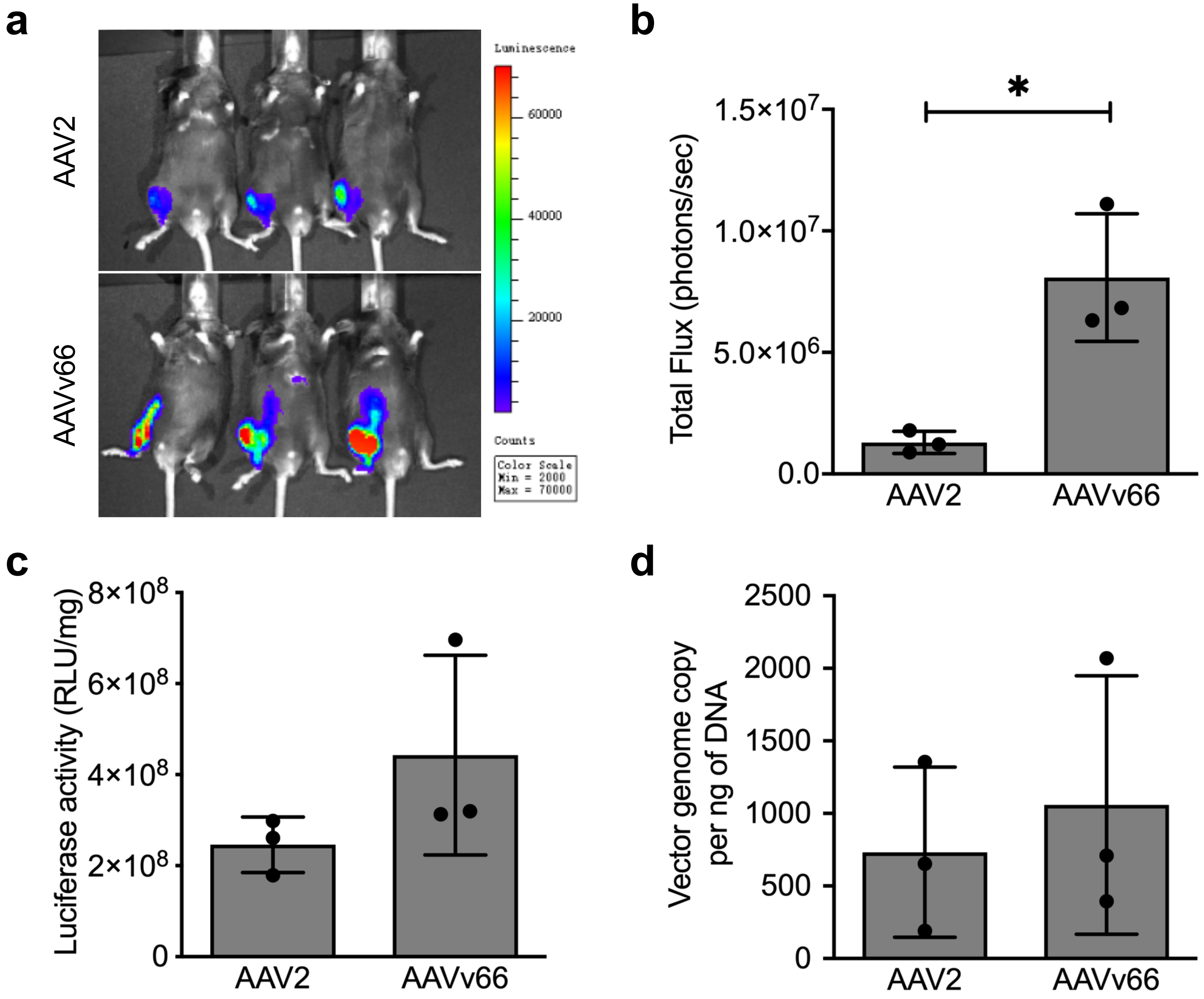

Supplementary Figure 6. Intramuscular administration of AAVv66 vector shows transduction of muscle. Intramuscular injection of AAVv66 into the tibialis anterior resulted in very little difference in transduction capacity when compared with the transduction of AAV2. AAV2-*CB6-PI-Fluc* or AAVv66-*CB6-PI-Fluc* (4.0E10 GC per mouse) was injected into mice by intramuscular administration into one hindlimb (tibialis anterior). **(a)** After 14 days, mice were injected with luciferin substrate intraperitoneally and imaged. Scale bar represents luminescence (flux, photons/sec) **(b)** Total flux of the injected hindlimb in acquired images (**panel a**) was recorded. Tissues were harvested and assayed for luciferase activity **(c)** and vector genome abundance by qPCR **(d)**. Values represent mean ±SD, n = 3 mice/group, *p <0.05 by two-tailed Student's t test.

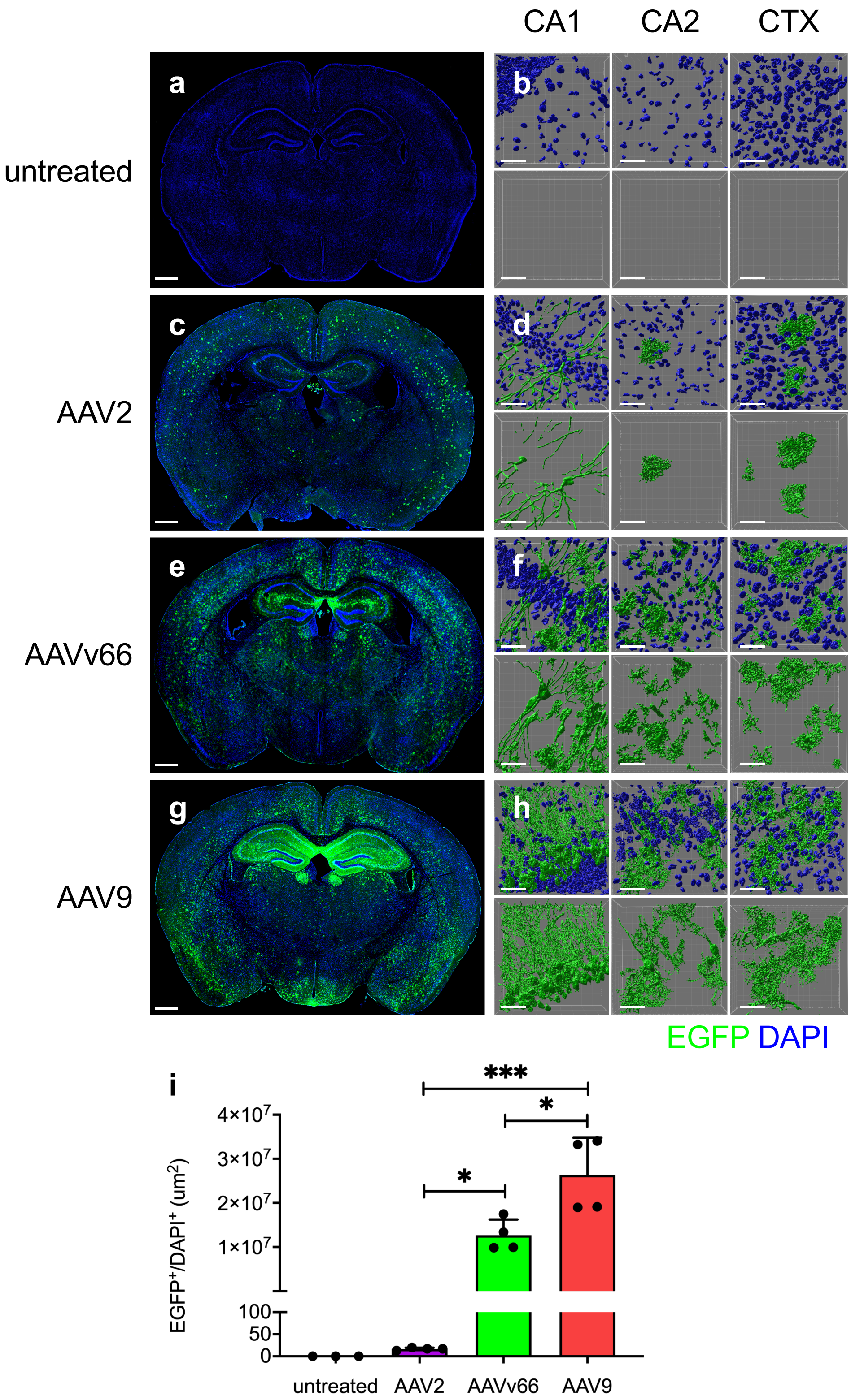

Supplementary Figure 7. AAVv66-*EGFP* vectors transduce the CNS following IV injection in neonatal mice. Immunofluorescence microscopy of cryo-sectioned brains from mice treated at P0-P1 via facial-vein injection with AAV vectors packaging the *EGFP* transgene. Shown are comparisons of EGFP distributions in untreated animals **(a, b),** by AAV2 **(c, d),** AAVv66 **(e, f),** or AAV9 **(g, h). (a, c, e, and g)** representative coronal sections of treated mouse brains stained with αGFP antibody and DAPI. Scale bars = 700 µm. **(b, d, f, and h)** 63X-magnified 3D-rendered cells in sub-anatomical regions (hippocampus (CA1 and CA2) and cortex (CTX)). Scale bars = 20 µm. **(i)** Quantification of EGFP positive surfaces shows that AAVv66 transduces mouse brains about two-fold less than what is achieved by AAV9. Data presented as the mean ±SD, n = 4 animals/group from one experiment, ****p <0.0001, One-way ANOVA followed by Tukey's multiple comparisons test.

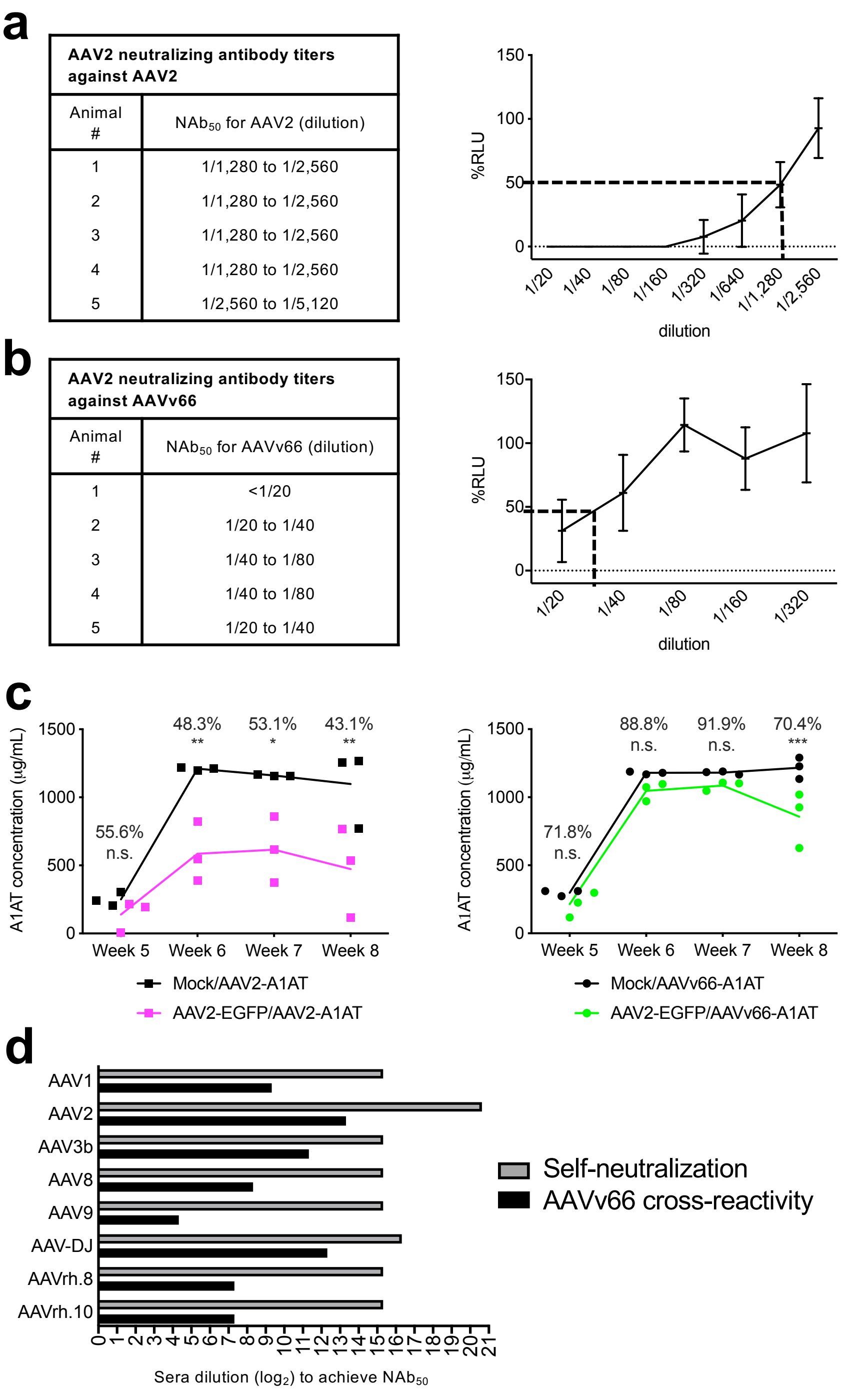

Supplementary Figure 8. AAV2 NAbs do not inhibit transduction by AAVv66 vectors. Mice were intramuscularly administrated by AAV2-*CB6-PI-EGFP* vector (1E11 GC per mouse). Four weeks after administration, sera were collected for testing neutralizing antibody (NAb) titers against AAV2 or AAVv66 infection. NAb_{50} values for **(a)** AAV2 and **(b)** AAVv66 are defined as the titer dilution that can block 50% of the total transduction achievable by vector packaged with the LacZ reporter gene. Left, NAb summaries of individual animals tested. Right, transduction efficiencies were plotted against various serum dilutions. Values represent mean ±SD. Dashed lines indicate mean NAb_{50} serum titers. **(c)** After the four-week period, mice were intramuscularly administrated with AAV2-*CB7-CI-hA1AT* or AAVv66-*CB7-CI-hA1AT* (1E11 GC per mouse) on the contralateral hindlimb. Serum A1AT levels were measured by ELISA at weeks 5, 6, 7, and 8. Values represent mean ±SD, n = 3/group, n.s. = not significant, *p <0.05, **p <0.01, and ***p <0.001 by two-way ANOVA on cross-sectional data points. **(d)** Rabbit anti-AAV serum cross-reactivity. Rabbit antisera raised against AAV serotypes was tested for NAb to AAVv66 (black bars) versus the homologous AAV serotype (grey bars) to assess relative cross reactivity. Log_2 values represent highest antibody dilution to achieve 50% inhibition of transduction.

a

AAVv66 neutralizing antibody titers against AAV2	
Animal #	NAb50 for AAV2 (dilution)
1	1/160 to 1/320
2	1/160 to 1/320
3	1/1,280 to 1/2,560
4	1/1,280 to 1/2,560
5	1/1,280 to 1/2,560

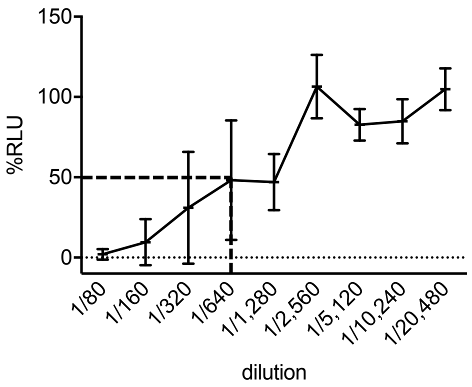

b

AAVv66 neutralizing antibody titers against AAVv66	
Animal #	NAb50 for AAVv66 (dilution)
1	1/640 to 1/1,280
2	1/2,560 to 1/5,120
3	1/2,560 to 1/5,120
4	1/2,560 to 1/5,120
5	1/2,560 to 1/5,120

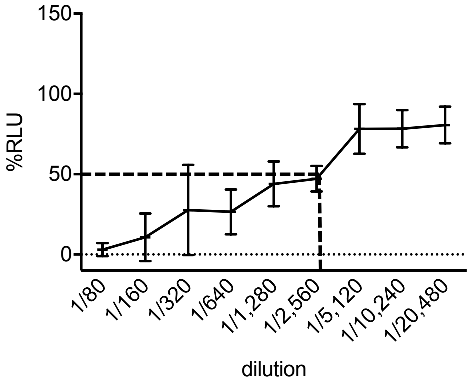

Supplementary Figure 9. AAVv66 NAbs can inhibit the transduction of AAVv66 and AAV2 vectors *in vitro*. Mice were intramuscularly administrated by AAVv66-*CB6-PI-EGFP* vector (1E11 GC per mouse). Four weeks after administration, sera were collected for testing neutralizing antibody (NAb) titers against AAV2 or AAVv66 infection. NAb_{50} values for **(a)** AAV2 and **(b)** AAVv66 are defined as the titer dilution that can block 50% of the total transduction achievable by vector packaged with the LacZ reporter gene. Left, NAb summaries of individual animals tested. Right, transduction efficiencies were plotted against various serum dilutions. Values represent mean ±SD. Dashed lines indicate mean NAb_{50} serum titers.

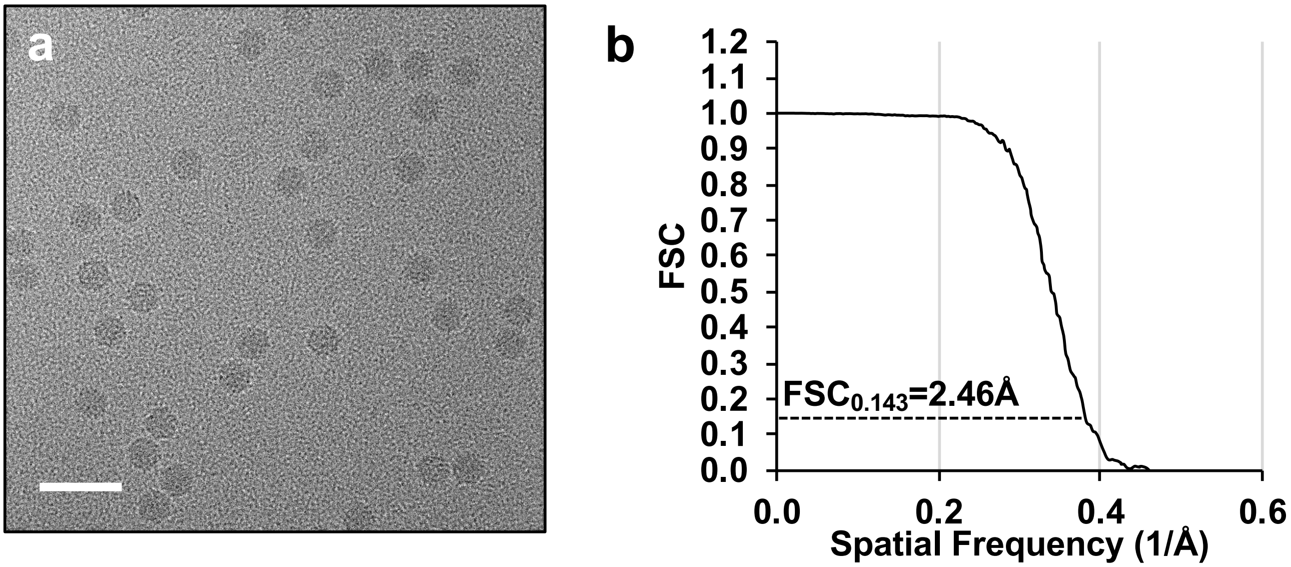

Supplementary Figure 10. Cryo-EM primary metrics, map reconstruction, and model generation of AAVv66. (a) Representative micrograph image (Titan Krios 300 KeV) of AAVv66. The scale bar represents 100 Å. **(b)** Fourier shell correlation for even and odd particles (FSC_part) for AAVv66.

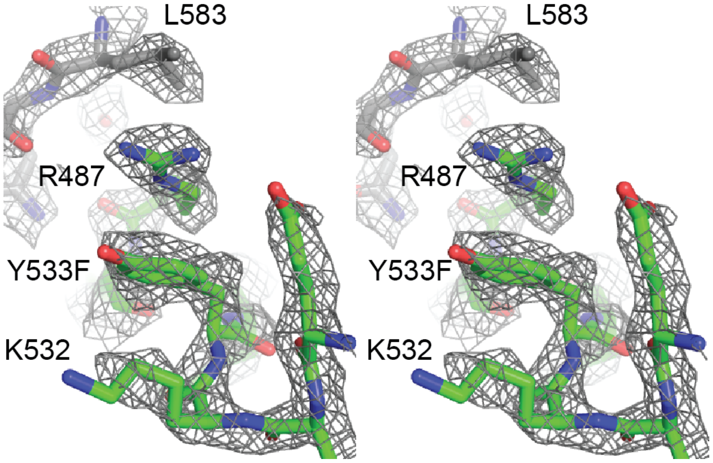

Supplementary Figure 11. Stereo image of AAVv66 electron density. A representative region of reconstructed and beta-factor sharpened electron density map of AAVv66, represented as gray wire mesh and displayed in stereo with the atomic model, as in **Figure 5c**.

a

AAVv66 Structural Comparisons, RMSD$_{C\text{-}\alpha}$ (Å)

Region:	VP3	VR I	VR II	VR III	VR IV	VR V	VR VI	VR VII	VR VIII	HI Loop	VR IX
Residues:	217-735	262-268	326-330	380-388	449-468	487-504	525-541	544-556	579-594	436-454	704-711
AAV2 (1LP3)	0.46	0.79	1.14	0.47	0.67	0.46	0.43	0.50	0.51	0.45	0.40
AAV3b (3KIC)	0.76	1.68	1.05	0.52	2.73	0.89	0.68	0.63	0.75	0.60	0.87

b

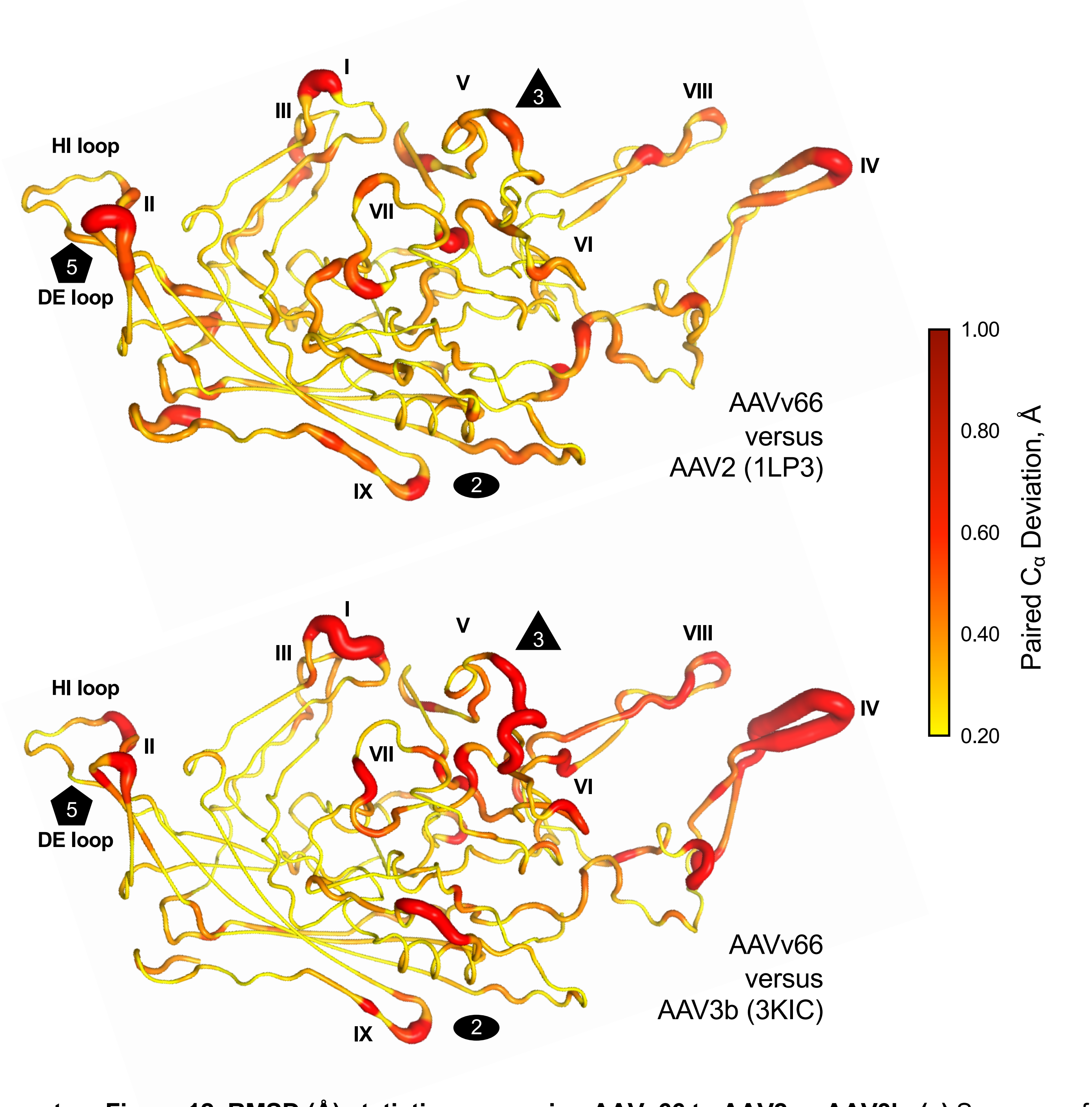

Supplementary Figure 12. RMSD (Å) statistics comparing AAVv66 to AAV2 or AAV3b. (a) Summary of the total and regional RMSD (Å) between AAVv66 and AAV2 (1LP3) or AAV3b (3KIC) measured across all alpha-carbon pairs indicated (AAV2 numbering) calculated by the *rms_cur* function within PyMOL. **(b)** Full capsid structures of AAV2, 3b, and AAVv66 were aligned through optimized fit within the cryo-EM density map of AAVv66. Using a custom script within PyMOL, the distance values (Å) between individual alpha-carbon pairs for either AAV2 (upper) or AAV3b (lower) were quantitatively transformed for representation as both color and radial thickness for the corresponding residues of AAVv66. The color scale bar represents the RMSD of C_α atoms of each residues pairs in Å units.
