# Supplementary material for: Structural characterization of a novel human adeno-associated virus capsid with neurotropic properties
Source: Nat Commun. 2020 Jun 30;11:3279. doi: 10.1038/s41467-020-17047-1 (PMC7327033; doi:10.1038/s41467-020-17047-1)
Supplement: Supplementary file 3 — Reporting Summary [file 41467_2020_17047_MOESM3_ESM.pdf]

nature research

Corresponding author(s): Guangping Gao
Andrei A. Korostelev
Phillip W.L. Tai

Last updated by author(s): May 3, 2020

Reporting Summary

Nature Research wishes to improve the reproducibility of the work that we publish. This form provides structure for consistency and transparency in reporting. For further information on Nature Research policies, see Authors & Referees and the Editorial Policy Checklist .

Statistics

For all statistical analyses, confirm that the following items are present in the figure legend, table legend, main text, or Methods section.

n/a	Confirmed	
☐	☒	The exact sample size (n) for each experimental group/condition, given as a discrete number and unit of measurement
☐	☒	A statement on whether measurements were taken from distinct samples or whether the same sample was measured repeatedly
☐	☒	The statistical test(s) used AND whether they are one- or two-sided<br>*Only common tests should be described solely by name; describe more complex techniques in the Methods section.*
☐	☒	A description of all covariates tested
☐	☒	A description of any assumptions or corrections, such as tests of normality and adjustment for multiple comparisons
☐	☒	A full description of the statistical parameters including central tendency (e.g. means) or other basic estimates (e.g. regression coefficient) AND variation (e.g. standard deviation) or associated estimates of uncertainty (e.g. confidence intervals)
☐	☒	For null hypothesis testing, the test statistic (e.g. F, t, r) with confidence intervals, effect sizes, degrees of freedom and P value noted<br>*Give P values as exact values whenever suitable.*
☒	☐	For Bayesian analysis, information on the choice of priors and Markov chain Monte Carlo settings
☒	☐	For hierarchical and complex designs, identification of the appropriate level for tests and full reporting of outcomes
☒	☐	Estimates of effect sizes (e.g. Cohen's d, Pearson's r), indicating how they were calculated

Our web collection on statistics for biologists contains articles on many of the points above.

Software and code

Policy information about availability of computer code

Data collection

Single molecule real-time (SMRT) sequencing was performed on the RSII platform using standard analysis software (smrtanalysis v2.2.0).
Cryo-EM data was collected using SerialEM (version 3.6, http://bio3d.colorado.edu/SerialEM/) on a Titan Krios electron microscope (FEI) operating at 300 kV and equipped with a Gatan Image Filter (GIF) and a K2 Summit direct electron detector (Gatan Inc.) using 0.5-2.2 µm underfocus.
Brain section images were acquired on a Leica SP8 Lightning High Resolution Confocal (Leica Microsystems, Wetzlar, Germany). Global brain images (10X tiled brain sections) and high-magnification images (63X region specific areas) were collected at the same intensity and exposure thresholds for each respective magnification.

Data analysis

Reads were mapped using the BWA-MEM algorithm. Reads were processed through InDelFixer (https://github.com/cbg-ethz/InDelFixer) to remove single nucleotide insertions and deletions that may result from error-prone PCR or sequencing error. In order to consider only unique capsid sequences and to rule out low-confidence variants, we performed de novo assembly (Geneious R9) on the filtered reads to cluster reads with 99% of sequence similarity.
Predicted amino acid sequences were aligned using the MUSCLE algorithm, iterating until convergence was achieved. PhyML (version 3.3. 20190909) was then used to generate the phylogenetic tree using default parameters from within SeaView (version 4.7) and then visualized via the Interactive Tree of Life online tool.
Particles were automatically picked within cisTEM (characteristic and maximum radius: 130 and 140 Å) (version 1.0.0-beta) . Initial reference for alignment was generated from all particles using the Ab initio 3D reconstruction function. This reference and all particles were iteratively refined using auto refine to obtain a 2.95-Å resolution map as determined from the FSC_part cutoff at 0.143. One round of per-particle CTF refinement in manual mode improved map resolution to 2.62 Å. Lastly, one round of beam tilt refinement and reconstruction improved map resolution to 2.46 Å. 3D classification did not improve the maps. The final map was B-factor sharpened by applying a B-factor of -32.92 Å2 using the PHENIX (version 1.14-3260) auto sharpen function.
Variant residues were modeled using PyMOL (The PyMOL Molecular Graphics System, Version 2.0 Schrödinger, LLC.). The resulting AAVv66 model, was refined using PHENIX against the cryo-EM map. Real-space simulated annealing and B-factor refinement in PHENIX resulted in a stereochemically optimal model. The model was inspected, and figures were prepared using PyMOL.
Luciferase activity in live animals was quantified using the IVIS SpectrumCT imaging platform (Perkin Elmer) with 1 min exposures. Image

acquisition was performed using Living Image software (version 4.7).
Graphs and statistical calculations were conducted in Prism 8 (GraphPad Software, Inc., San Diego, CA).
For high-magnification images, 40-50 z-stack steps were collected at a 0.29 z-size. Analysis was performed using Imaris 9.3 Software (Bitplane Inc., Zurich, Switzerland). Each image was 3D rendered and thresholds were manually established. To ensure consistency, non-biased 3D rendering of total sub-anatomical EGFP volumes colocalized with DAPI volumes and cell type-specific stains were used as proxies for cellular counts and the number of positively transduced cells. Percent quantifications of the different cell types within each ipsilateral sub-anatomical region was conducted, followed by percentage quantification of each cell type. Percent transduction was determined by normalizing colocalized EGFP volume to total volume of cell type-specific staining within each region. Per cell-specific stain, n=3 mice were analyzed.

For manuscripts utilizing custom algorithms or software that are central to the research but not yet described in published literature, software must be made available to editors/reviewers. We strongly encourage code deposition in a community repository (e.g. GitHub). See the Nature Research guidelines for submitting code & software for further information.

Data

Policy information about availability of data

All manuscripts must include a data availability statement. This statement should provide the following information, where applicable:

- Accession codes, unique identifiers, or web links for publicly available datasets
- A list of figures that have associated raw data
- A description of any restrictions on data availability

Capsids and vector constructs described in this report will be made available upon request. The model and map of AAVv66 will be available from the RCSB Protein Data Bank PDB: 6U3Q and the Electron Microscopy Database: EMD-20630, respectively. The nucleic acid sequence for AAVv66 has been deposited at NCB Genbank as 2252913. The PDB ID: 1LP3 does not belong to us and is related to DOI: 10.1073/pnas.162250899, 10.2210/pdb1lp3/pdb.

Field-specific reporting

Please select the one below that is the best fit for your research. If you are not sure, read the appropriate sections before making your selection.

☒ Life sciences ☐ Behavioural & social sciences ☐ Ecological, evolutionary & environmental sciences

For a reference copy of the document with all sections, see nature.com/documents/nr-reporting-summary-flat.pdf

Life sciences study design

All studies must disclose on these points even when the disclosure is negative.

Sample size	Generally, sample sizes were calculated on the assumption that a 30% difference in the parameters measured would be considered biologically significant with an estimate of sigma of 10-20% of the expected mean. Alpha and Beta were set to the standard values of .05 and 0.8, respectively. Statistical analyses reported use either Student's t-test or one-way ANOVA where applicable.
Data exclusions	No data were excluded
Replication	Each experimental test/study is supported by replicate analysis (2-3 times). All attempts at replication were successful.
Randomization	Mouse injection groups described were non-random, but were aged-matched across experiments.<br>Only one tissue from human origin is described in this study. Therefore, no randomization for allocation or control for covariates for this study was needed.
Blinding	Data collections were blinded for the initial steps of the study. Animal studies were carried out by multiple investigators to mitigate bias: J.L. and L.R. performed mouse injections, who were blinding to the treatment groups by coding; A.L. prepared the tissues and executed the immunofluorescence workflows; and P.W.L.T. and D.G. performed data analysis and interpretations . In addition, all study parameters are based on quantitative data and therefore did not require blinding to rule out investigator bias.

Reporting for specific materials, systems and methods

We require information from authors about some types of materials, experimental systems and methods used in many studies. Here, indicate whether each material, system or method listed is relevant to your study. If you are not sure if a list item applies to your research, read the appropriate section before selecting a response.

Materials & experimental systems

n/a	Involved in the study	
☐	☒	Antibodies
☐	☒	Eukaryotic cell lines
☒	☐	Palaeontology
☐	☒	Animals and other organisms
☐	☒	Human research participants
☒	☐	Clinical data

Methods

n/a	Involved in the study	
☒	☐	ChIP-seq
☒	☐	Flow cytometry
☒	☐	MRI-based neuroimaging

Antibodies

Antibodies used

anti-NeuN, 1:1000, EMD Millipore MAB377; anti-Gfap, 1:500, EMD Millipore MAB360; anti-Olig2, 1:200, Abcam ab109186; anti-Iba1, 1:1000, Wako Chemicals NC9288364; anti-GFP, 1:800, Invitrogen A11122;
Anti-Human Alpha 1-Antitripsin, 1:1000 Developed in rabbit [A-0409 Sigma];
Conjugate: Goat anti-α1-anti-trypsin-HRP antibody, 1:5,500 [Fitzgerald (lot#:15181)];
anti-mouse, Invitrogen A32744;
anti-rabbit, Invitrogen A32740

Validation

anti-NEUN, 1:1000, EMD Millipore MAB377;
application:Anti-NeuN Antibody, clone A60 detects level of NeuN and has been published and validated for use in FC, IC, IF, IH, IH (P), IP and WB.
anti-GFAP, 1:500, EMD Millipore MAB360;
Anti-Glial Fibrillary Acidic Protein Antibody, clone GA5 detects level of Glial Fibrillary Acidic Protein & has been published & validated for use in IC, IH, IH(P) & WB with more than 65 product citations.
anti-OLIG2, 1:200, Abcam ab109186;
Host species: Rabbit
Application:Suitable for: WB, IHC-P, IHC-Fr
anti-IBA1, 1:1000, Wako Chemicals NC9288364;
Species: Human, rat
Application: ICC; 50ug
anti-GFP, 1:800, Invitrogen A11122
Species: Rabbit
Application: 1672 articles have cited and used this antiboy for WB, IF, IHC, etc.

Eukaryotic cell lines

Policy information about cell lines

Cell line source(s)

HEK293 cells were either obtained from ATCC or Invitrogen

Authentication

Initial authentications were provided by vendor/manufacturer
https://www.atcc.org/products/all/CRL-1573.aspx
https://www.thermofisher.com/us/en/home/technical-resources/cell-lines/2/cell-lines-detail-176.html
No further authentication was performed.

Mycoplasma contamination

Cell lines were negative for myoplasma.

Commonly misidentified lines
(See ICLAC register)

HEK293. These are the standard cell lines for AAV production and verification (Gao, G. and M. Sena-Esteves (2012). "Introducing Genes into Mammalian Cells: Viral Vectors." Molecular Cloning: A Laboratory Manual vol. 2.: 1209-1313.)

Animals and other organisms

Policy information about studies involving animals; ARRIVE guidelines recommended for reporting animal research

Laboratory animals

Mus musculus, C57BL/6j, male, neonates and 6-8 weeks. Mice were kept on a 12 hrs light / 12 hrs darkness light cycle, at 70-74 F with humidity at 35%-46%. Mice were fed normal chow (ISO-pro 300 Irradiated Diets (#5P76).

Wild animals

No wild animals were used in the study.

Field-collected samples

No field collected samples were used in the study.

Ethics oversight

All animal procedures described in this study were approved by the UMass Medical School Animal Care and Use Committee.

Note that full information on the approval of the study protocol must also be provided in the manuscript.

Human research participants

Policy information about studies involving human research participants

Population characteristics	A pancreatic neoplasm sample was acquired from a 71-year-old female patient following tumorectomy and pathology of the tissue by frozen section examination and intraoperative frozen section diagnosis (Department of Oncology, West China Hospital of Sichuan University, Chengdu, China).
Recruitment	N/A
Ethics oversight	For DNA extraction from human tissues, approval was obtained from West China Hospital Institution Ethics Committee. Patient consent or waiver was not required for this study, since the specimen was de-identified in abidance to the HIPAA Privacy Rule and coded such that the identity of the individual was not able to be readily ascertained.

Note that full information on the approval of the study protocol must also be provided in the manuscript.
